# Supplementary material for: Community volunteer participation and its determinants during respiratory infectious disease outbreaks in China: A cross-sectional study across multiple provinces
Source: PLoS One. 2025 Aug 25;20(8):e0330838. doi: 10.1371/journal.pone.0330838 (PMC12377566; doi:10.1371/journal.pone.0330838)
Supplement: S1 Table — (S1_Table.PDF) [file pone.0330838.s001.pdf]

S1 Table. Multicollinearity Assessment: Variance Inflation Factors for Independent Variables in Logistic Regression Model.

Variable	Variance Inflation Factors
**Sex**	1.032
**Age**	1.270
**Household registration**	1.053
**Marital status**	1.193
**Educational attainment**	1.227
**Employment**	1.177
**Political affiliation**	1.195
**Frequency of volunteering before 2020**	1.197
