# Supplementary material for: Community volunteer participation and its determinants during respiratory infectious disease outbreaks in China: A cross-sectional study across multiple provinces
Source: PLoS One. 2025 Aug 25;20(8):e0330838. doi: 10.1371/journal.pone.0330838 (PMC12377566; doi:10.1371/journal.pone.0330838)
Supplement: S2 Table — (S2_Table.PDF) [file pone.0330838.s002.pdf]

S2 Table. Adjusted Odds Ratios for Volunteer Participation with Multiple Comparison Correction Using Bonferroni Method.

Variable	COR (95% CI)	AOR (95% CI)	P-value	Bonferroni Corrected P-value
**Men**	Reference	Reference		
**Women**	1.330 (1.026, 1.723)*	0.874 (0.630, 1.213)	0.421	
**Age 18-34**	Reference	Reference		
**Age 35-44**	1.445 (1.056, 1.979)*	0.819 (0.503, 1.333)	0.421	
**Age 45-70**	1.641 (1.193, 2.257)**	1.263 (0.773, 2.062)	0.352	
**Urban**	Reference	Reference		
**Rural**	0.776 (0.596, 1.010)	0.793 (0.563, 1.115)	0.182	
**Married/living with partner**	Reference	Reference		
**Single/divorced/widowed**	0.355 (0.267, 0.473)***	0.603 (0.389, 0.935)*	0.024	
**Primary/junior secondary**	Reference	Reference		
**High-school/technical college/associate**	1.190 (0.654, 2.164)	1.845 (0.869, 3.919)	0.111	
**Bachelor's degree or higher**	1.762 (0.996, 3.118)	2.576 (1.21, 5.484)*	0.014	
**Employment: Other**	Reference	Reference		
**Employment: Healthcare staff**	4.886 (2.800, 8.526)***	2.476 (1.313, 4.670)**	0.005	<0.05

**Employment: Student**	0.774 (0.522, 1.148)	1.584 (0.780, 3.216)	0.204	
**Employment: Street/community staff**	8.712 (5.451, 13.926)***	4.315 (2.559, 7.276)***	<0.001	<0.001
**Political affiliation: unaffiliated**	Reference	Reference		
**Communist Party member**	4.505 (3.108, 6.530)***	1.679 (1.069, 2.640) *	0.025	
**Democratic party member**	3.506 (0.407, 30.195)	1.005 (0.098, 10.301)	0.997	
**Communist Youth League member**	0.701 (0.478, 1.029)	0.547 (0.280, 1.069)	0.078	
**Frequency of volunteering before 2020 (5-level scale)**	3.521 (2.967, 4.167)***	3.021 (2.513, 3.636)***	<0.001	<0.001

* $p < 0.05$; ** $p < 0.01$; *** $p < 0.001$.
COR = crude odds ratio; AOR = adjusted odds ratio (mutually adjusted for all covariates).
