# Supplementary material for: Community volunteer participation and its determinants during respiratory infectious disease outbreaks in China: A cross-sectional study across multiple provinces
Source: PLoS One. 2025 Aug 25;20(8):e0330838. doi: 10.1371/journal.pone.0330838 (PMC12377566; doi:10.1371/journal.pone.0330838)
Supplement: Attachment — Submitted filename: point_to_point response2.pdf [file pone.0330838.s005.pdf]

Dear PLOS ONE Editorial Team,

Thank you very much for your detailed and thoughtful feedback on our manuscript submission. We greatly appreciate the opportunity to revise and improve our materials in alignment with PLOS ONE's editorial standards. Please find our point-to-point responses below addressing each of your comments with care:

Point-to-point Response on 6/5:

1. Please upload a copy of the original ethics approval letter in Chinese, as required by question 1 of the Human Subjects Research Checklist. Also keep the provided translated copy visible in the file inventory.

Response: Thank you for your careful review and for bringing this to our attention. We have uploaded the original ethics approval letter in Chinese as requested. Kindly note that there is a discrepancy in the signature dates between the Chinese and English versions. This occurred because the Chinese version was the initial official approval document issued by the Peking University Ethics Office, and the English version was subsequently obtained for publication purposes. Both versions were formally authorized and signed by the same ethics committee at Peking University. We hope this clarification is helpful, and we sincerely appreciate your guidance.

Point-to-point Response on 5/30:

1. We notice that you Cover Letter are incomplete details missing title of Manuscript Please update your Cover Letter with title. thanks

Response: Thank you very much for pointing this out. We have now updated the cover letter to include the full title of the manuscript at the beginning. We appreciate your careful review and guidance.

2. Please complete all items on the Human Participants Research Checklist that are relevant for your submission, by following this link: http://journals.plos.org/plosone/s/file?id=dc11/PLOSOne_Human_Subjects_Research_Checklist.docx (Contact us at plosone@plos.org if you cannot access the document.) There may be overlap between the checklist items and other queries

listed below; please address any duplicated queries both in your response email and on the checklist itself. Upload the completed Human Participants Research Checklist as file type "Other" when you re-submit your manuscript. This document is for internal journal use only and will not be published if your article is accepted. The requested information will help us to assess whether your submission complies with PLOS ONE's policies and adheres to applicable reporting standards. Note that your manuscript may be rejected if you provide incomplete or inadequate responses to the checklist questions and that changing the 'Section/Category' of your article does not affect this requirement.

Response: Thank you for the reminder. We have carefully reviewed the Human Participants Research Checklist and completed all applicable items. The completed form has been uploaded as requested, under the file name PLOSOne_Human_Subjects_Research_Checklist.pdf and categorized as "Other" in the submission system. We appreciate your efforts to uphold rigorous ethical standards.

3. Please provide additional details regarding participant consent. In the ethics statement in the Methods and online submission information, please ensure that you have specified what type you obtained (for instance, written or verbal, and if verbal, how it was documented and witnessed). If your study included minors, state whether you obtained consent from parents or guardians. If the need for consent was waived by the ethics committee, please include this information.

Once you have amended this/these statement(s) in the Methods section of the manuscript, please add the same text to the "Ethics Statement" field of the submission form (via "Edit Submission").

Response: Thank you for this important observation. In response, we have clarified the consent process in the Methods section (lines 133–136) by adding the following statement: "The study protocol was approved by the Peking University Institutional Review Board (IRB00001052-22171). Electronic written informed consent for participation and publication was obtained from all participants through the online survey platform, immediately prior to initiating the questionnaire." We have also updated the "Ethics Statement" field in the online submission form accordingly.

As noted in the manuscript (lines 124–125), only adult participants aged 18–70 were eligible; therefore, no minors were included in the study. We are grateful for your attention to ethical details and your guidance in ensuring full transparency.
