# Supplementary material for: Community volunteer participation and its determinants during respiratory infectious disease outbreaks in China: A cross-sectional study across multiple provinces
Source: PLoS One. 2025 Aug 25;20(8):e0330838. doi: 10.1371/journal.pone.0330838 (PMC12377566; doi:10.1371/journal.pone.0330838)
Supplement: Attachment — Submitted filename: Reviewer_comments.docx [file pone.0330838.s006.docx]

**1. Is the manuscript technically sound, and do the data support the conclusions?**

**Yes**, the manuscript is technically sound. The study is well-conceived and uses a large, geographically diverse sample (N = 1,023) from five provinces in China. The authors applied appropriate cross-sectional survey methods and used logistic regression to identify factors associated with community volunteer participation during respiratory infectious disease outbreaks.

The findings—particularly the associations between participation and factors like education, occupation, political affiliation, and previous volunteering—are well supported by the data. The conclusions are logical and appropriately nuanced, with limitations acknowledged.

**2. Has the statistical analysis been performed appropriately and rigorously?**

**Yes**, the statistical analyses are sound and appropriately rigorous:

- **Descriptive statistics** clearly outline participant characteristics, volunteer roles, motivations, and barriers.
- **Binary logistic regression** models estimate both crude and adjusted odds ratios, accounting for potential confounders.
- The **sample size** is adequate for the analyses conducted, and stratification across regions enhances generalizability.
- Statistical significance was appropriately set at α = 0.05, and confidence intervals were reported for all key results.

There are no signs of inappropriate statistical tests or overinterpretation of results.

**3. Have the authors made all data underlying the findings in their manuscript fully available?**

**Partially.** The data are available **upon reasonable request** from the corresponding author, which complies with some journal policies but **does not meet PLOS ONE’s full open data requirement**, which asks for unrestricted access unless there are specific legal or ethical constraints.

The authors should be encouraged to deposit de-identified data in a public repository (e.g., Dryad, Figshare) or clearly justify restrictions.

**4. Is the manuscript presented in an intelligible fashion and written in standard English?**

**Yes.** The manuscript is clearly written in fluent and professional English. It is logically structured, with coherent flow from the background through the discussion. Key terms are defined, and acronyms are used consistently. There are very few, if any, grammatical or typographical errors, and technical language is appropriate for a public health audience.

**5. Review Comments to the Author**

**General Comments:**

This is a valuable and timely study that contributes to understanding community-based epidemic response mechanisms. The high volunteer participation rates and identification of enabling and inhibiting factors offer useful guidance for public health policy and emergency planning.

**Strengths:**

- **Strong design and regional coverage**, enhancing the relevance of the findings.
- **Clear operational definitions** of volunteer activity types and outcomes.
- **Use of validated frameworks** (e.g., Volunteer Functions Inventory).
- **Robust discussion** that connects findings to policy and literature.

**Areas for Improvement:**

1. **Data Availability Compliance:** Please consider making anonymized data publicly available in line with PLOS ONE’s open data policies or provide a more detailed justification for data restrictions.
2. **Clarify Limitations Further:** The authors note limitations well, but more elaboration on how web-based recruitment might bias toward more educated participants could strengthen the discussion.
3. **Ethical Clarity:** The revised ethics statement is acceptable, but consider adding a short note confirming how data privacy was protected given the online format (e.g., IP anonymization, server security, etc.).
4. **Role Descriptions Could Be Expanded:** The categorization of volunteer activities is appropriate, but adding more granular examples could help contextualize certain findings (e.g., what constitutes “psychosocial support”?).
5. **Discussion of Non-Participation:** Further interpretation of why nearly 34% did not participate, despite broad support for volunteering might enrich understanding and policy implications.

**Final Recommendation:**

**Minor Revision Recommended.**

This paper is scientifically rigorous and policy-relevant. With modest improvements in data sharing and further clarification in the discussion, it would make a strong contribution to literature on community resilience in public health emergencies.
