# Supplementary material for: Community volunteer participation and its determinants during respiratory infectious disease outbreaks in China: A cross-sectional study across multiple provinces
Source: PLoS One. 2025 Aug 25;20(8):e0330838. doi: 10.1371/journal.pone.0330838 (PMC12377566; doi:10.1371/journal.pone.0330838)
Supplement: Attachment — Submitted filename: review.docx [file pone.0330838.s007.docx]

This manuscript presents a timely and well-structured cross-sectional study exploring the prevalence and determinants of community volunteering during respiratory infectious disease outbreaks in China. The topic is highly relevant, the methodology is sound, and the findings offer practical implications for public health preparedness. The paper is largely well-written, though certain sections would benefit from improved clarity, better theoretical integration, and expanded contextualization.

1. Abstract
   - The abstract is well-balanced but does not report any effect sizes. Consider briefly mentioning key odds ratios for impactful variables.
2. Introduction
   - Explicitly explain why the five provinces (Beijing, Guangdong, Heilongjiang, Hubei, Yunnan) were chosen—e.g., diversity in region, epidemic severity, or urban-rural balance.
   - The manuscript references China’s national volunteer guidelines and social emergency deployments, which is helpful. However, it would be beneficial to clarify whether there is any formal integration between these national volunteer frameworks and health emergency systems (e.g., were community volunteers mobilized via formal government channels during COVID-19?).
3. Methods
   - Clarify how missing data (if any) were handled.
   - State whether multicollinearity among independent variables was assessed.
   - For the logistic regression, explain whether model fit was evaluated.
4. Results
   - While the manuscript thoroughly examines various sociodemographic and motivational factors, income—often considered a key determinant of volunteerism—was not included in the analysis. It would strengthen the study’s transparency and theoretical framing to briefly explain why this variable was omitted.
   - To enhance interpretability, I suggest adding a column in Table 1 that displays the proportion of participants who volunteered versus those who did not, stratified by each characteristic (e.g., sex, age group, education level). This would allow readers to more intuitively assess how volunteering varies across demographic groups prior to reviewing the regression results in Table 5.
5. Discussion
   - The manuscript frequently refers to “value expression” as the primary motivational factor for volunteering, but the term remains abstract throughout the text.
   - Consider differences in volunteer infrastructure between urban and rural settings when discussing implementation strategies.
6. General
   - Please ensure consistency in the number of decimal places used for odds ratios (ORs) and adjusted odds ratios (AORs) between the main text and tables. For example, some values are reported to two decimal places in the text, while others appear with three in the tables.
   - Ensure consistent use of terminology (e.g., healthcare vs health‑care).
