# Supplementary material for: Community volunteer participation and its determinants during respiratory infectious disease outbreaks in China: A cross-sectional study across multiple provinces
Source: PLoS One. 2025 Aug 25;20(8):e0330838. doi: 10.1371/journal.pone.0330838 (PMC12377566; doi:10.1371/journal.pone.0330838)
Supplement: Attachment — Submitted filename: Response_to_Reviewers.docx [file pone.0330838.s008.docx]

Dear Dr. Rajaguru and reviewers,

Thank you so much for your thorough review and constructive feedback on our manuscript. We greatly appreciate the opportunity to strengthen our work through your insightful comments. We have carefully addressed each point raised and have made corresponding revisions to improve the manuscript, particularly the transparency and clarity of our Methods and Discussion sections. Below, we provide detailed responses to each comment in **blue**, and all line numbers referenced correspond to the clean version of the revised manuscript:

**Response to Editor Comments**

**Editorial Requirements**

1. **Please ensure that your manuscript meets PLOS ONE's style requirements, including those for file naming. The PLOS ONE style templates can be found at** [**https://journals.plos.org/plosone/s/file?id=wjVg/PLOSOne_formatting_sample_main_body.pdf**](https://journals.plos.org/plosone/s/file?id=wjVg/PLOSOne_formatting_sample_main_body.pdf) **and** [**https://journals.plos.org/plosone/s/file?id=ba62/PLOSOne_formatting_sample_title_authors_affiliations.pdf**](https://journals.plos.org/plosone/s/file?id=ba62/PLOSOne_formatting_sample_title_authors_affiliations.pdf)

Response: Thank you very much for providing the style templates. We have thoroughly updated our manuscript to comply with PLOS ONE's formatting requirements, including proper file naming conventions, author and affiliation formatting, subsection titles, reference styling, and body text formatting according to the provided templates.

2. **We note that you have indicated that there are restrictions to data sharing for this study. For studies involving human research participant data or other sensitive data, we encourage authors to share de-identified or anonymized data. However, when data cannot be publicly shared for ethical reasons, we allow authors to make their data sets available upon request. For information on unacceptable data access restrictions, please see** [**http://journals.plos.org/plosone/s/data-availability#loc-unacceptable-data-access-restrictions**](http://journals.plos.org/plosone/s/data-availability#loc-unacceptable-data-access-restrictions)**.**

**Before we proceed with your manuscript, please address the following prompts:**

**a) If there are ethical or legal restrictions on sharing a de-identified data set, please explain them in detail (e.g., data contain potentially identifying or sensitive patient information, data are owned by a third-party organization, etc.) and who has imposed them (e.g., a Research Ethics Committee or Institutional Review Board, etc.). Please also provide contact information for a data access committee, ethics committee, or other institutional body to which data requests may be sent.**

**b) If there are no restrictions, please upload the minimal anonymized data set necessary to replicate your study findings to a stable, public repository and provide us with the relevant URLs, DOIs, or accession numbers. Please see http://www.bmj.com/content/340/bmj.c181.long for guidelines on how to de-identify and prepare clinical data for publication. For a list of recommended repositories, please see https://journals.plos.org/plosone/s/recommended-repositories. You also have the option of uploading the data as Supporting Information files, but we would recommend depositing data directly to a data repository if possible.**

**Please update your Data Availability statement in the submission form accordingly.**

Response: We really appreciate your guidance on the data sharing requirements. We have uploaded our minimal de-identified dataset to the figshare data repository and updated our Data Availability statement accordingly.

The statement now reads: “The minimal de-identified dataset used in the current study is available from [https://figshare.com/articles/dataset/Data_for_the_study_Community_volunteer_participation_and_its_determinants_during_respiratory_infectious_disease_outbreaks_in_China_a_cross-sectional_study_across_multiple_provinces_/29602721?file=56387153].”

3. **In the online submission form, you indicated that the datasets used during the current study are available from the corresponding author upon reasonable request.**

**All PLOS journals now require all data underlying the findings described in their manuscript to be freely available to other researchers, either 1. In a public repository, 2. Within the manuscript itself, or 3. Uploaded as supplementary information.**

**This policy applies to all data except where public deposition would breach compliance with the protocol approved by your research ethics board. If your data cannot be made publicly available for ethical or legal reasons (e.g., public availability would compromise patient privacy), please explain your reasons on resubmission and your exemption request will be escalated for approval.**

Response: As noted in the response to comment#2, we made our de-identified dataset publicly available through the figshare repository to meet PLOS ONE’s data availability requirements. The dataset is now accessible to other researchers for verification and further analysis.

**Additional Editor Comments**

4. **The Methods section requires revision to improve transparency and clarity in several key areas, including sampling limitations, participant recruitment flow, and variable validation.**

**Study Design: The use of a non-probability sampling method (quota sampling via an online platform) introduces potential selection bias. This is especially important given that internet access, digital literacy, and willingness to participate in online surveys can vary significantly across different socioeconomic groups. This limitation should be explicitly acknowledged in the manuscript's limitations section.**

Response: We completely agree with this important feedback. Given the post-pandemic context and associated constraints, an online survey approach was selected to enhance sample representativeness and ensure timely data collection. We have now explicitly acknowledged this limitation in our Discussion section under limitations.

We now have: “… our use of non-probability quota sampling via an online platform may have introduced selection bias, as internet access, digital literacy, and willingness to participate in online surveys can vary significantly across socioeconomic groups. This may also have resulted in a sample with higher educational attainment and digital engagement than the general population, potentially limiting the generalizability of our findings to all demographic groups” (lines 383-387).

5. **Participants and Sampling: The sample size of approximately 200 respondents per province may be insufficient to detect regional differences or interaction effects unless justified with a power calculation or sensitivity analysis.**

Response: Thank you for this valuable point. We acknowledge that our study was primarily designed to examine overall patterns of volunteer participation rather than detect regional differences. The target sample size was determined using the formula N = [Z^2^_1-α/2_P(1-P)]/d², where α = 0.05, Z_1-α/2_ = 1.96, and the acceptable margin of error d = 0.10. Based on previous research estimating 30% prevalence of relevant outcomes during the COVID-19 pandemic, a minimum of 81 participants per province was required. Our achieved sample of approximately 200 respondents per province exceeded this threshold and was adequate for our primary objective of identifying factors associated with volunteer participation overall.

We have clarified this in our revised Methods section by adding: “We used the formula N = [Z^2^_1-α/2_P(1-P)]/d², where α = 0.05, Z_1-α/2_ = 1.96, the acceptable margin of error d = 0.10, and the 30% prevalence of relevant outcomes during the COVID-19 pandemic to estimate the target sample size. As a result, we got a minimum required sample of 81 participants per province [16]” (lines 114-117) and “…leaving 1,023 valid cases (effective response rate 99.8 %). This satisfied our sample size calculation requirement of 81 participants per province (405 in total)” (lines 135-136). We also acknowledged the limitation regarding regional comparisons in the Discussion: “Fifth, our study was designed primarily to examine overall patterns of volunteer participation rather than detect regional differences, and the sample size of approximately 200 respondents per province may be insufficient for robust regional comparisons or interaction effect analyses” (lines 389-392).

6. **The authors should clarify whether statistical weights were applied to better align the sample with national demographic distributions.**

Response: We did not apply statistical weights in our analysis. Our quota sampling approach was designed to approximate national distributions for key demographic variables (sex, age, and household registration).

We updated out Methods-Statistical analysis section to clarify this: “No statistical weights were applied to the data, as our quota sampling approach was designed to approximate national demographic distributions” (lines 191-192).

In our Methods- Recruitment and sampling section, we had described: “We used quota sampling to approximate the national population distribution reported in the Seventh National Census in China. Quotas were set for sex (men 51 %, women 49 %), age (18–39 years 40 %, 40–70 years 60 %), and Chinese household registration (urban 65 %, rural 35 %)” (lines 120-122) to demonstrate that our sampling design aligned with national demographic distributions to ensure that our sample composition closely mirrors the target population characteristics.

7. **Furthermore, the rationale for selecting only five provinces should be provided, along with an explanation for excluding other populous or epidemiologically important provinces such as Sichuan and Shanghai.**

Response: Thank you for requesting this clarification. We have now provided a detailed rationale for province selection in our Methods section. The five provinces were selected based on multiple criteria: (1) regions with the highest cumulative COVID-19 cases from each of the five major geographical regions (east, west, south, north, and central China), (2) economic development diversity, and (3) geographical distribution to represent different regions of mainland China.

We added: “The selection of survey provinces considered multiple criteria including selecting areas with the highest cumulative COVID-19 case counts before November 2022 from each of the five major geographical regions (northern, southern, eastern/central, western, and north‑eastern), economic development diversity, and geographical distribution to ensure representative coverage of mainland China [16]” to our Method section (lines 110-114).

8. **Statistical Analysis: The analysis section lacks details on whether multidisciplinary among independent variables was assessed prior to performing multivariable logistic regression.**

Response: Thank you for this important methodological question. We conducted comprehensive multicollinearity assessment using variance inflation factors (VIF) prior to performing our multivariable logistic regression analysis. All VIF values ranged from 1.032 to 1.270, well below the commonly accepted threshold of 5, indicating acceptable levels of multicollinearity among independent variables.

We have added this information to our Statistical Analysis section: “Prior to conducting multivariable logistic regression, we assessed multicollinearity among independent variables using variance inflation factors (VIFs). All VIF values ranged from 1.032 to 1.270 (S1 Table 1), well below the threshold of 5, indicating acceptable levels of multicollinearity” (lines 187-190).

We also added Supplementary 1 Table 1 to show the exact numbers of VIFs.

9. **Additionally, the manuscript does not describe how missing data were handled—whether through list-wise deletion, imputation, or another method.**

Response: Thanks a lot for requesting clarification on this important aspect of our methodology. Our study design eliminated missing data through mandatory response requirements implemented via the Wenjuanxing platform. We set the platform's system configuration to require participants completing all questionnaire items before submission was permitted, resulting in complete data for all study variables without the need for imputation or deletion methods.

We have clarified this in our Data Collection section of the Methods: “We set the online questionnaire platform to require mandatory completion of all questions, with participants unable to submit responses without answering every item, thereby ensuring complete data collection for all study variables” (lines 136-139).

10. **The authors should also clarify how the significance level was adjusted, if at all, to account for multiple comparisons. Overall, the statistical analysis section should be revised to include these methodological details and ensure that appropriate statistical procedures were applied.**

Response: We really appreciate this comment. We improved our analysis by adding Bonferroni correction for multiple comparisons in our logistic regression. After correction, only the most robust predictors maintained statistical significance: occupation (street/community staff: AOR = 4.315, 95% CI: 2.559-7.276, p<0.001; healthcare staff: AOR = 2.476, 95% CI: 1.313-4.670, p<0.05) and frequency of volunteering before 2020 (AOR = 3.021, 95% CI: 2.513-3.636, p<0.001). Variables such as education, marital status, and political affiliation, while showing meaningful effect sizes in uncorrected analysis, did not survive the conservative correction threshold.

We have made the following additions to address this:

- Methods-Statistical analysis section: “To account for multiple comparisons, Bonferroni correction was applied with appropriate adjustment of significance levels” (lines 192-194).
- Results section: “After Bonferroni correction for multiple comparisons, the following variables remained statistically significant: occupation (street/community staff: AOR = 4.32, 95% CI: 2.56-7.28, p<0.001; healthcare staff: AOR = 2.48, 95% CI: 1.31-4.67, p<0.05) and frequency of volunteering before (AOR = 3.02, 95% CI: 2.51-3.64, p<0.001) (S1 Table 2)” (lines 239-43). We also added Supplementary 1 Table 2 to show the exact numbers.
- Discussion section: “The application of Bonferroni correction for multiple comparisons provided a more conservative assessment of these associations, with only the most robust predictors, occupation (street/community staff and healthcare personnel) and prior volunteer experience, maintaining statistical significance after correction. Variables such as education level, marital status, and political affiliation, while demonstrating meaningful effect sizes in uncorrected analysis, did not survive the stringent correction threshold. These findings suggest that occupation and prior experience represent the most reliable predictors of volunteer participation, while other demographic factors may reflect borderline associations that warrant validation through larger-scale studies or alternative analytical approaches” (lines 342-350).

11. **Also recommended to include the significance and policy implications of the study.**

Response: Thank you very much for this recommendation. We have expanded our Discussion section to better highlight the policy significance and practical implications of our findings in lines 359-379:

“Collectively, these results have significant implications for emergency preparedness and community resilience systems in China and similar contexts. The robustness of occupation and prior experience as predictors suggests that emergency management agencies should prioritize targeted recruitment strategies focusing on healthcare workers, community staff, and individuals with established volunteer networks. Developing volunteer registries that maintain contact with experienced volunteers could facilitate rapid mobilization during future health emergencies. The 2024 revision of the Emergency Response Law establishes that social organizations and volunteers should participate in emergency response work under unified government command, though specific implementation guidelines are still under development [31]. Our findings on occupation and prior volunteer experience as key predictors can inform these implementation guidelines, particularly by providing evidence-based criteria for targeted recruitment and volunteer classification systems during health emergencies. The current proportion of residents (34%) who did not participate in volunteer activities, despite broad community support for volunteering, suggests important opportunities for expanding engagement. Our barrier analysis indicates that addressing time constraints, skill development needs, and infection control concerns could potentially convert non-participants into active volunteers, underscoring the importance of flexible volunteer opportunities and robust safety protocols to maximize community participation during health emergencies. Implementation strategies should also consider differences between urban and rural volunteer infrastructure, with urban areas benefiting from technology-enhanced platforms while rural communities may require emphasis on informal networks and locally adapted roles.”

**Response to Reviewer#1 Comments**

**General Comments:**

**This is a valuable and timely study that contributes to understanding community-based epidemic response mechanisms. The high volunteer participation rates and identification of enabling and inhibiting factors offer useful guidance for public health policy and emergency planning.**

**Strengths:**

- **Strong design and regional coverage, enhancing the relevance of the findings.**
- **Clear operational definitions of volunteer activity types and outcomes.**
- **Use of validated frameworks (e.g., Volunteer Functions Inventory).**
- **Robust discussion that connects findings to policy and literature.**

Response: We are very grateful for your acknowledgment that this study contributes meaningfully to understanding community-based epidemic response mechanisms and offers useful guidance for public health policy and emergency planning.

**Areas for Improvement:**

1. **Data Availability Compliance: Please consider making anonymized data publicly available in line with PLOS ONE’s open data policies or provide a more detailed justification for data restrictions.**

Response: Thank you for emphasizing the importance of open data policies. We have addressed this concern by making our anonymized dataset publicly available through the figshare repository, in complete compliance with PLOS ONE’s data availability requirements.

We have updated our Data Availability statement to: “The minimal de-identified dataset used in the current study is available from [https://figshare.com/articles/dataset/Data_for_the_study_Community_volunteer_participation_and_its_determinants_during_respiratory_infectious_disease_outbreaks_in_China_a_cross-sectional_study_across_multiple_provinces_/29602721?file=56387153].”

2. **Clarify Limitations Further: The authors note limitations well, but more elaboration on how web-based recruitment might bias toward more educated participants could strengthen the discussion.**

Response: We really appreciate this important suggestion regarding web-based recruitment bias. We have expanded our limitations section to provide more detailed elaboration on how our recruitment method might have been biased toward more educated participants.

We have enhanced our limitations section with: “Third, our use of non-probability quota sampling via an online platform may have introduced selection bias, as internet access, digital literacy, and willingness to participate in online surveys can vary significantly across socioeconomic groups. This may also have resulted in a sample with higher educational attainment and digital engagement than the general population, potentially limiting the generalizability of our findings to all demographic groups” (lines 383-387).

3. **Ethical Clarity: The revised ethics statement is acceptable, but consider adding a short note confirming how data privacy was protected given the online format (e.g., IP anonymization, server security, etc.).**

Response: Thank you for this important clarification regarding data privacy protection in online formats. We have added details about our privacy protection measures to enhance transparency in our ethical procedures.

We have added to our Methods section: “The online platform implemented comprehensive privacy protection measures including IP address anonymization, secure server protocols, and encrypted data transmission to ensure participant confidentiality throughout the data collection process” (lines 124-127).

4. **Role Descriptions Could Be Expanded: The categorization of volunteer activities is appropriate, but adding more granular examples could help contextualize certain findings (e.g., what constitutes “psychosocial support”?).**

Response: We appreciate this suggestion for providing more granular examples of volunteer activities. We have expanded our role descriptions to better contextualize our findings and help readers understand the specific nature of volunteer contributions.

We have enhanced our Methods section by adding more detailed examples: “The type of service was classified into five predefined categories adapted from the framework of Mak et al. [5] and current community practice: 1) nucleic‑acid testing assistance (e.g. QR‑code registration, queue management, and temperature monitoring), 2) registration of residents’ entry to and exit from the community (including visitor screening and resident identification verification), 3) temperature or health‑code screening at community checkpoints (including symptom monitoring and health status verification), 4) delivery of supplies to quarantined households (including food, medication, and essential items), and 5) dissemination of epidemic‑prevention information (including distributing educational materials, conducting health education sessions, and providing psychosocial support to community members)” (lines 149-158).

5. **Discussion of Non-Participation: Further interpretation of why nearly 34% did not participate, despite broad support for volunteering might enrich understanding and policy implications.**

Response: This is an excellent suggestion that enriches our understanding of volunteer engagement patterns. We have added discussion regarding the 34% non-participation rate and its policy implications.

We have added to our Discussion section: “The current proportion of residents (34%) who did not participate in volunteer activities, despite broad community support for volunteering, suggests important opportunities for expanding engagement. Our barrier analysis indicates that addressing time constraints, skill development needs, and infection control concerns could potentially convert non-participants into active volunteers, underscoring the importance of flexible volunteer opportunities and robust safety protocols to maximize community participation during health emergencies” (lines 370-376).

**Response to Reviewer#2 Comments**

**General Comments:**

**This manuscript presents a timely and well-structured cross-sectional study exploring the prevalence and determinants of community volunteering during respiratory infectious disease outbreaks in China. The topic is highly relevant, the methodology is sound, and the findings offer practical implications for public health preparedness. The paper is largely well-written, though certain sections would benefit from improved clarity, better theoretical integration, and expanded contextualization.**

Response: We really appreciate your positive assessment of our cross-sectional study and acknowledge your suggestions for enhanced clarity and better theoretical integration, which we have systematically addressed below.

1. **Abstract**

**The abstract is well-balanced but does not report any effect sizes. Consider briefly mentioning key odds ratios for impactful variables.**

Response: Thank you for this excellent suggestion to include effect sizes in the abstract. We have revised our abstract to include key odds ratios for the most impactful variables to enhance the informativeness of our findings.

We have updated our abstract to include: “Higher odds of participation were observed among respondents who held at least a bachelor's degree (AOR = 2.58, 95% CI: 1.21-5.48), worked in community roles (AOR = 4.32, 95% CI: 2.56-7.28) or healthcare roles (AOR = 2.48, 95% CI: 1.31-4.67), were Communist Party members (AOR = 1.68, 95% CI: 1.07-2.64), or had volunteered regularly before 2020 (AOR = 3.02, 95% CI: 2.51-3.64), while single/divorced/widowed individuals had lower odds of participation (AOR = 0.60, 95% CI: 0.39-0.94) (all p < 0.05)” (lines 50-56).

2. **Introduction**

- **Explicitly explain why the five provinces (Beijing, Guangdong, Heilongjiang, Hubei, Yunnan) were chosen—e.g., diversity in region, epidemic severity, or urban-rural balance.**

Response: We really appreciate this comment. To better clarify our sampling rationale, we added relevant content in our Methods section as: “The selection of survey provinces considered multiple criteria including selecting areas with the highest cumulative COVID-19 case counts before November 2022 from each of the five major geographical regions (northern, southern, eastern/central, western, and north‑eastern), economic development diversity, and geographical distribution to ensure representative coverage of mainland China [16]” (lines 110-114).

- **The manuscript references China’s national volunteer guidelines and social emergency deployments, which is helpful. However, it would be beneficial to clarify whether there is any formal integration between these national volunteer frameworks and health emergency systems (e.g., were community volunteers mobilized via formal government channels during COVID-19?).**

Response: Thank you very much for this important comment regarding formal integration. In the Introduction section, we have added: “During the COVID-19 pandemic, community volunteers were mobilized through formal government channels (including neighborhood committees and street offices) and informal community networks, which created a hybrid volunteer mobilization system that leveraged existing social infrastructure while expanding capacity through grassroots engagement. Although formal integration mechanisms are not yet fully established in China, the legal framework for volunteer integration in emergencies continues to evolve based on these practical experiences” (lines 71-77).

Additionally, we enhanced our Discussion section to address the current regulatory landscape and related implications of our study: “The 2024 revision of the Emergency Response Law establishes that social organizations and volunteers should participate in emergency response work under unified government command, though specific implementation guidelines are still under development [31]. Our findings on occupation and prior volunteer experience as key predictors can inform these implementation guidelines, particularly by providing evidence-based criteria for targeted recruitment and volunteer classification systems during health emergencies” (lines 364-370).

3. **Methods**

Response: Thank you for these important methodological clarifications. We have addressed all three concerns comprehensively below:

- **Clarify how missing data (if any) were handled.**

Response: For missing data handling, we have added: “We set the online questionnaire platform to require mandatory completion of all questions, with participants unable to submit responses without answering every item, thereby ensuring complete data collection for all study variables” (lines 136-139).

- **State whether multicollinearity among independent variables was assessed.**

Response: For multicollinearity assessment, we have added: “Prior to conducting multivariable logistic regression, we assessed multicollinearity among independent variables using variance inflation factors (VIFs). All VIF values ranged from 1.032 to 1.270 (S1 Table 1), well below the threshold of 5, indicating acceptable levels of multicollinearity” (lines 187-190).

We also added Supplementary 1 Table 1 to show the exact numbers of VIFs.

- **For the logistic regression, explain whether model fit was evaluated.**

Response: For model fit evaluation, we have added: “Model fit was assessed using Nagelkerke R²” (lines 190-191) in the Methods section, and “This model demonstrated good fit (Nagelkerke R² = 0.446, explaining 44.6% of the variance)” (lines 228-229) in the Results section.

4. **Results**

- **While the manuscript thoroughly examines various sociodemographic and motivational factors, income—often considered a key determinant of volunteerism—was not included in the analysis. It would strengthen the study’s transparency and theoretical framing to briefly explain why this variable was omitted.**

Response: We really appreciate this suggestion for enhancing the transparency. For income variable omission, we have added to our Methods section: “Income was not included as a variable due to cultural sensitivity concerns in Chinese contexts, where individuals may be reluctant to accurately report income levels. Employment category was thus included as a proxy measure to capture relevant socioeconomic status differences” (lines 164-167).

- **To enhance interpretability, I suggest adding a column in Table 1 that displays the proportion of participants who volunteered versus those who did not, stratified by each characteristic (e.g., sex, age group, education level). This would allow readers to more intuitively assess how volunteering varies across demographic groups prior to reviewing the regression results in Table 5.**

Response: Thank you very much for this valuable suggestion to enhance interpretability. We originally had these columns in Table 5, but your comment helped us recognize their value for intuitive assessment. We have moved the volunteer participation columns (showing n(%) for volunteered and did not volunteer) to Table 1, stratified by each demographic characteristic, allowing readers to assess volunteering patterns across groups before reviewing the regression results.

5. **Discussion**

- **The manuscript frequently refers to “value expression” as the primary motivational factor for volunteering, but the term remains abstract throughout the text.**

Response: Thanks a lot for this important suggestion! For value expression clarification, we have added: “Value expression, as conceptualized in the Volunteer Functions Inventory, refers to individuals’ motivation to act on personally important values such as humanitarianism, altruism, and social responsibility. In our context, this manifested as volunteers' desire to help others, fulfill social obligations, and contribute to community well-being during the health crisis [9, 10]” (lines 268-271).

- **Consider differences in volunteer infrastructure between urban and rural settings when discussing implementation strategies.**

Response: We are grateful for this comment, and we have added “Implementation strategies should also consider differences between urban and rural volunteer infrastructure, with urban areas benefiting from technology-enhanced platforms while rural communities may require emphasis on informal networks and locally adapted roles” (lines 376-379) in the discussion section.

6. **General**

- **Please ensure consistency in the number of decimal places used for odds ratios (ORs) and adjusted odds ratios (AORs) between the main text and tables. For example, some values are reported to two decimal places in the text, while others appear with three in the tables.**
- **Ensure consistent use of terminology (e.g., healthcare vs health‑care).**

Response: Thank you for these important formatting and consistency observations. We have systematically reviewed and standardized all numerical reporting and terminology throughout the manuscript. We have ensured consistent reporting of odds ratios to two decimal places throughout both text and tables, and we also standardized the use of “healthcare” (rather than “health-care”) consistently across the manuscript.
